# Kernel principal components based cascade forest towards disease identification with human microbiota

**DOI:** 10.1186/s12911-021-01705-5

**Published:** 2021-12-23

**Authors:** Jiayu Zhou, Yanqing Ye, Jiang Jiang

**Affiliations:** 1 National University of Defense Technology, Changsha, China; 2 Consulting Center for Strategic Assessment, Academy of Military Sciences, Beijing, China; 3 Naval Submarine Academy, Qingdao, China

**Keywords:** Human microbiota, Supervised classification, Kernel principal components, Cascade forest, Disease identification

## Abstract

**Background:**

Numerous pieces of clinical evidence have shown that many phenotypic traits of human disease are related to their gut microbiome, i.e., inflammation, obesity, HIV, and diabetes. Through supervised classification, it is feasible to determine the human disease states by revealing the intestinal microbiota compositional information. However, the abundance matrix of microbiome data is so sparse, an interpretable deep model is crucial to further represent and mine the data for expansion, such as the deep forest model. What’s more, overfitting can still exist in the original deep forest model when dealing with such “large p, small n” biology data. Feature reduction is considered to improve the ensemble forest model especially towards the disease identification in the human microbiota.

**Methods:**

In this work, we propose the kernel principal components based cascade forest method, so-called KPCCF, to classify the disease states of patients by using taxonomic profiles of the microbiome at the family level. In detail, the kernel principal components analysis method is first used to reduce the original dimension of human microbiota datasets. Besides, the processed data is fed into the cascade forest to preliminarily discriminate against the disease state of the samples.

**Results:**

The proposed KPCCF algorithm can represent the small-scale and high-dimension human microbiota datasets with the sparse feature matrix. Systematic comparison experiments demonstrate that our method consistently outperforms the state-of-the-art methods with the comparative study on 4 datasets.

**Conclusion:**

Despite sharing some common characteristics, a one-size-fits-all solution does not exist in any space. The traditional depth model has limitations in the biological application of the unbalanced scale between small samples and high dimensions. KPCCF distinguishes from the standard deep forest model for its excellent performance in the microbiota field. Additionally, compared to other dimensionality reduction methods, the kernel principal components analysis method is more suitable for microbiota datasets.

## Background

The human microbiota is made up of about 100 trillion microbial cells. Compared to 10 trillion humanoid cells in our body, microbiota provides many missing features of human biology [1]. The content and number of gut microbes keep a dynamic balance during their hosts’ evolution, and microbes also assist their host to maintain normal physiological functions [2, 3]. There are numerous clinical studies exploring the association between microbiome and phenotype, aiming to identify differentially abundant taxa between health and disease [4], including inflammation [5], obesity [6–9], autism [10, 11], immune system diseases [12], neurological diseases [13] and cancer [14–16]. Recent advances in sequencing technologies have made it feasible to profile the microbiome via metagenomic sequencing, which is a technique to extract DNA from environmental samples [17]. Human microbiota genomics cooperative research programs have been launched internationally in recent years, such as the European Metagenomics of the Human Intestinal Tract [18] and the Human Microbiome Project [19]. These programs aim to understand the gut microbiota of healthy individuals through large-scale sequencing and use this as a reference to study the intestinal tract under disease conditions.

Biology classifies and names various taxa of organisms according to different levels, normally including Domain (d), Kingdom (k), Phylum (p), Class (c), Order (o), Family (f), Genus (g), and Species (s). At present, the classification of diseases by intestinal microbes is mainly based on the genus level [20]. A category at a higher level integrates multiple lower-level categories. As a result, the higher the level, the fewer sample categories can be classified. Moreover, higher-level categories are easier to obtain. Due to microorganisms themselves being very rich at the genus level, the established “sample-feature” matrix tends to be so sparse leading to unnecessary biological detection and calculation. If we can get good identification results from a higher level in meta-genome data, it will be more beneficial to be applied in the real application. Attempting to improve the performance of the dataset during prediction, our work applies the microbiome data at the family level as the diagnosis basis.

Using machine learning algorithms to identify highly complex and unknown patterns in datasets (such as human microbiota) is of great value [1]. It has been demonstrated that several existing supervised classifiers, such as Random Forests (RFs) and Support Vector Machine (SVMs) [21], can be effectively used to classify and predict the disease based on microbiota population. However, because of inconsistent individual studies and the lack of standardized data analysis methods, the accuracy of classifying and predicting diseases through the human intestinal microbiome is still unsatisfactory. Enhancing the complexity of an algorithm by deepening the network, increases not only the number of computing functions but also the degree of its embedding. [22] published an article, and the concept of “Deep Learning” (DL) was officially proposed. DL is a high-level abstraction algorithm that uses multiple complex structures to represent multiple nonlinear changes [23]. Deep Neural Networks (DNNs) have been widely exploited recently for meta-genomic association studies [24, 25], meta-genomic classification [26, 27], and disease diagnose [28, 29]. Large training data is necessary for DNNs to realize good performance, which may not be possible in small-scale datasets like biology and medical science. For example, almost all CNN faces over-fitting problems due to the limitation of data volume and the increase of training parameters. That is, the magnitude of the training set does not match the complexity of the model, and the weight learning iterations are overtraining, fitting the noise in the training data, and the non-representative features in the training examples. Recently, a Deep Forest (DF) model called gcForest was proposed by Zhou and Feng, which is an ensemble of ensembles decision tree method and performs excellently in many experiments [30, 31]. The interpretable tree structure can solve the problem of non-differentiable. Additionally, compared to the time-consuming parameter adjustment, gcForest is far more efficient due to fewer hyper-parameters.

In the gcForest model, a multi-grained scanning is conducted first to get its corresponding transformed feature representation. Sliding windows are used to scan the low-dimension features, and differently grained feature vectors will be generated by using multiple sizes of sliding windows. In the following, the instances extracted from the same size of windows are used to train the first grade of a cascade forest, containing completely-random tree forest and random forest. Random forest is an integrated model of random trees, introducing randomness to encourage diversity. While for the completely-random tree forest, it selects and assigns features completely randomly. The class vectors are generated and concatenated as transformed features.

However, the scanning model in gcForest can only consider the original sequence, which will lead to features disturbing for the unknown relationship between two adjacent features. The microbiota datasets are too sparse and contain lots of 0 values in many flora features. When the training sets are put into a multi-grained scanning package, due to the not yet clear complicated relationships between each microbiota, it can extract representative new features sometimes but others not. Thus, the standard DF model still faces overfitting and ensemble diversity challenges when dealing with such “large p, small n” biology data. Many researchers have been exploring how to improve the DF algorithm of identification for special field [27, 32, 33]. Features are the key to determining similarity measurements and classification predictions. To highlight some useful information and suppress the useless, it is necessary to reduce the input features. The original datasets can be transformed at the beginning of the algorithm to adapt to subsequent depth learning [34]. The affinity network model was put forward to learn from a limited number of training examples and generalizes well [35]. The kernel-based model can also offset the hyperplane by modifying the kernel function caused by the unbalanced data. [36] applied the kernel method to feature extraction and proposed kernel principal components analysis (kPCA) method. The experimental results show that kPCA can not only extract nonlinear features but also obtain better recognition results. KPCA is widely used in various fields such as industrial nonlinear process monitoring [37, 38] and image classification [39]. We systematically explored disease identification by utilizing the kPCA considering limited and unbalanced samples and a large number of features. To further improve the meta-genomic classification accuracy, we use the mixed data fused with associated metadata, such as gender, age, and other basic information as the diagnosis basis and fed them to the proposed model.

## Methods

The disease identification can be treated as a multi-class classification problem, and all the datasets we use here contain three categories. This section presents the datasets’ information and detailed procedures of the KPCCF method for disease identification. The four microbiota datasets used in our paper are introduced first. In the following subsection, the kernel principal components analysis method is applied to reduce the original dimension of the microbiota datasets. Then, we use cascade forests to preliminarily discriminate against the disease state of the sample with the reduced human gut microbiota. Finally, the overall procedure of KPCCF is detailedly present.

### Microbiota datasets

Sequencing technology can directly sequence microbial DNA, generating a large number of microbial sequencing data. According to the analysis object and experimental purpose, the research of meta-genomics can be basically divided into amplicon sequencing and meta-genomic complete sequencing. The former obtains the relative abundance and diversity level of each bacterial species to understand the composition and structure of the microbial community in the environment, including 16s rRNA, etc. The latter is the overall sequencing and analysis of all meta-genomic DNA, including Shotgun metagenomics, etc. Many people now use the above sequencing data to carry out prediction research [21, 24, 27].

MicrobiomeHD [40] is a standardized database of human gut microbiome studies on health and disease. This database includes publicly available 16s data from published case-control studies and their associated patient metadata. In this work, four datasets derived from MicrobiomeHD are used to verify the effects of the gut microbiome on the occurrence of different diseases in humans. The datasets we chose are related to four popular diseases, Clostridium Difficile Infection (CDI), Colorectal Cancer (CRC), Inflammatory Bowel Diseases (IBD), and Obesity (OB). CDI is the main cause of antibiotic-associated diarrhea. With the increase in its incidence rate, CDI has already become one of the most important public health problems that threaten human beings’ health. CRC, the world’s second-largest cancer, is malignant cancer caused by the accumulation of genetic mutations, which causes a massive proliferation and spread of more than 50%. IBD is caused by abnormal responses of the immune system of the genetically susceptible host to environmental factors, including Crohn’s disease (CD) and ulcerative colitis (UC). Different disease states occur under the combined action of environmental factors and intestinal microbes. OB measures are the incidence of overweight/obesity (OW/OB). Table 1 shows the detailed divisions of the used datasets. Specifically, in the cdi_schubert dataset [41], the samples consist of 93 *CDI*, 89 *nonCDI*, and 154 *H* samples, in which nonCDI represents patients with diarrhea who tested negative for CDI, CDI represents patients that suffer from CDI, and H represents the healthy samples. The crc_baxter dataset [15] consists of 120 *CRC*, 198 *adenoma*, and 172 controls, in which CRC represents tumor disease infection, adenoma signifies adenoma infection, and H denotes the healthy samples. In the ibd_papa [42] dataset, there are 24 *nonIBD*, 43 *UC*, and 23 *CD*, in which non-IBD controls are patients with gastrointestinal symptoms but no intestinal inflammation. While the ob_goodrich dataset [43] possess 185 *OB* (obesity), 336 *OW* (overweight) and 428 controls.

**Table 1:** Number of datasets samples and features

ID	Data sources	Disease label and sample size	f-level features	g-level features
1	cdi_schubert [41]	CDI(93), nonCDI(89), H(154)	80	198
2	crc_baxter [15]	CRC(120), H(172), adenoma(198)	93	255
3	ibd_papa [42]	nonIBD(24), UC(43), CD(23)	49	142
4	ob_goodrich [43]	OB(185), OW(336),H(428)	79	199

The datasets all come from real-world cases. Each dataset contains a metadata table, an OTU (Operational Taxonomic Units) table, and other related information. The metadata table involves various physical characteristics such as gender, age, and disease state of the patient. OTU is an operation classification unit that artificially groups sequences according to a certain degree of similarity. Since the microbiota community has no explicit relationship so far, there are many types of research carried out using RNA sequencing [33], DNA sequencing [34], and clinical images [39]. Some experiments choose OTU as an additional supplement nowadays. However, only a few related types of research use microbiota OTU data for research, and the results obtained were not ideal. Our paper only used the OTU table for prediction to mine the relationship between the patients and their microbiota. Thus, our results only generated by OTU are more competitive.

**Fig. 1:**
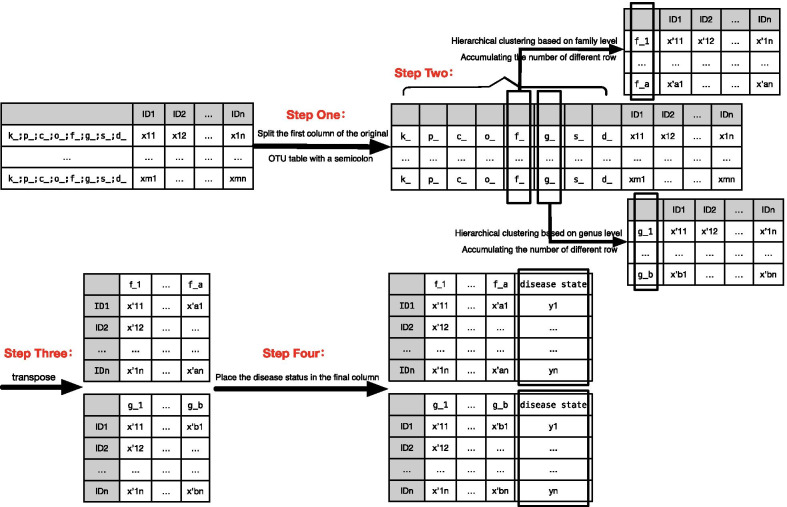
Introduction to the datasets. Firstly, split the first column of the original OTU table by a semicolon, and connect the split series expanding the columns of the original OTU table. Secondly, hierarchically cluster the microbiota, and accumulate different numbered samples. Thirdly, transpose the table obtained in the previous step. Fourthly, placing the disease state in the metadata set as the final column

To mine the microbiome data, the datasets need to be processed and converted into a sample-feature matrix first. The procedure of data processing is shown in Fig. 1. $Step\ One$, split the first column of the original OTU table by a semicolon, and connect the split series expanding the columns of the original OTU table. $Step\ Two$, according to the columns of the genus and the family level, the flora is hierarchically clustered, and the number of communities of different numbered samples is accumulated together. $Step\ Three$, transpose the table obtained in the previous step to a sample-microbiota features table. $Step\ Four$, place the disease state in the metadata set as the final column. The processed sample dataset is represented as a sample/feature dimension, and the last column is the annotation of the disease state.

### Kernel principal components based feature reduction

The number of training samples needs to grow exponentially with the feature dimension [44]. That is if *N* training samples are enough to cover the one-dimensional feature space, then $N^2$ samples are needed to cover the two-dimensional feature space of the same density, $N^3$ samples are needed to cover the three-dimensional feature space, and so on. From the very beginning, as the feature dimension increases, the performance of the classifier will gradually increase. However, after the number of features reaches a certain point, the prediction accuracy gradually decreases. Both redundant features (which can be derived from other features), and irrelevant features (which do not affect model training) are catastrophic for machine learning algorithms. Dimensional disaster always leads to weak generalization, so it is necessary to first reduce the dimension to avoid overfitting. Removing unrelated features can not only reduce the difficulty and speed of learning tasks but also enhance the understanding between features and eigenvalues.

Since it is unclear about the biological mechanism of action and the relationship between every microbiota population, directly eliminating the “useless” features may result in information omission. Therefore, we use the feature transformation method to reduce the dimension of data. During the features mapping from one-dimensional space to another, only the eigenvalues will change accordingly. Kernel Principal Components Analysis (kPCA) is a nonlinear extension of the Principal Components Analysis (PCA) algorithm. We use the kPCA method to reduce the intestinal microbiota characteristics dimension. The process of kPCA is to raise the original dimension data to new *k*-dimensional, and the final goal is to make the data linearly separable in the target dimension, which is the maximum separability of PCA. The kernel-based model can also offset the hyperplane by modifying the kernel function caused by the unbalanced data. By replacing the original data with a kernel function, it is possible to mine the nonlinear information contained in the datasets. It describes the correlation between multiple features and captures important information to achieve better results. What’s more, dimension reduction can also remove some noise and unnecessary details, and effectively speed up the training process.

We choose the kPCA method depending on the following considerations: (1) the calculation of the kernel function is independent of the feature dimension. The introduction of kernel function avoids the direct operation of high-dimensional feature space after transformation, greatly reducing the calculation amount and avoiding the “dimensionality disaster”. Some kernel functions, such as the RBF kernel, make the dimension of feature space infinite to improve the pattern classification or regression ability. (2) There is no need to know the form and parameters of the nonlinear transformation function. The calculation of kernel function in the original input space essentially implicitly corresponds to a high-dimensional nonlinear transformation function. The transformation overcomes the limitation of the nonlinear feature space dimension.

There are no obvious performance metrics to help choose the best kernel method and hyper-parameter values for kPCA, which is an unsupervised learning algorithm. We use the grid search method to select the kernel function and gamma values that will allow the task to perform optimally and get the best classification accuracy. There are many kinds of kernel functions, such as linear kernel functions, polynomial kernel functions, sigmoid kernel functions, and Gaussian kernel functions, etc. Gaussian kernel functions, also called Radial Basis Function (RBF), are the most commonly used.

**Fig. 2:**
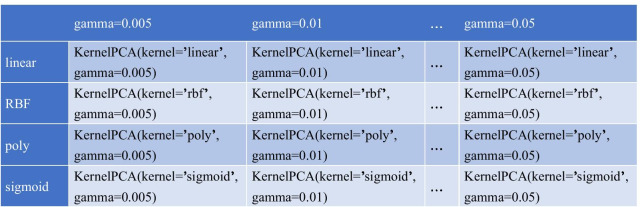
The parameters selection in grid search. We used four kernel functions, “linear”, “rbf”, “poly”, and ”sigmoid” for verification, and combined the information of the four datasets to verify the gamma as 0.005, 0.01, 0.02, 0.03, 0.04, 0.05, 0.06

Grid Search is a parameter tuning method through an exhaustive search. In the selection of all candidate parameters, it tries every possible combination of parameters through loop traversal and outputs the parameter combination gaining the best result. We used four commonly used kernel functions, “linear”, “rbf”, “poly”, and “sigmoid” for verification. The last three kernel functions all require a common parameter gamma. Gamma is equivalent to adjusting the complexity of the model. The higher the gamma value, the greater the model complexity, which may easily lead to overfitting. The default value of gamma is the reciprocal of the feature number. Due to the different characteristics of the datasets we used, we combined the information of the four data sets to verify the gamma as 0.005, 0.01, 0.02, 0.03, 0.04, 0.05, 0.06. Grid Search is a parameter tuning method through an exhaustive search. As shown in Fig. 2, in the selection of all candidate parameters, it tries every possible combination of parameters through loop traversal and outputs the parameter combination gaining the best result. In the end, a combination with the best result was selected: “kernel=rbf, gamma=0.05”. We used this as the parameter of the final experiment. The RBF kernel is presented as:

1 $$\begin{aligned} K(x,x')=exp\left( -\frac{\left\| x-x' \right\| _{2}^{2}}{2\sigma ^{2}}\right) \end{aligned}$$

where $\left\| x-x' \right\| _{2}^{2}$ is the squared Euclidean distance between two feature vectors, $\sigma$ is a free parameter. It can map the input data into infinite dimensions. An equivalent but the simpler definition is to set a new parameter $\gamma =\frac{1}{2\alpha ^{2}}$, then the expression can be expressed as:

2 $$\begin{aligned} K(x,x')=exp\left( -\gamma {\left\| x-x' \right\| _{2}^{2}}\right) \end{aligned}$$

The value of the RBF kernel ranges from 0 to 1, which is a similar metric representation and decreases as the distance increases. The feature space of the kernel has an infinite number of dimensions. For $\sigma =1$, its expansion is:

3 $$\begin{aligned} exp\left( -\frac{1}{2}\left\| x-x' \right\| _{2}^{2}\right) =\sum _{j=0}^{\infty }\frac{(x^{T}x')^j}{j!}exp\left( -\frac{1}{2}\left\| x \right\| _{2}^{2}\right) exp\left( -\frac{1}{2}\left\| x' \right\| _{2}^{2}\right) \end{aligned}$$

### Ensemble classification model of cascade forest

The main goal of the paper is to explore the relationship between microbes and disease occurrence based on community and quantity of intestinal microbiota. However, the abundance matrix data of the microbiome is too sparse with the small sample size even after appropriate dimensionality reduction. That is, most microbes are limited to a relatively small number of samples. A deep model is needed to represent and mine the data. The integrated cascade forest model is the ensemble of both breadth and depth of the traditional forest model.

Cascade forest is an ensemble of ensembles method, which is composed of random forests and completely-random tree forests in its structure. A completely-random tree forest randomly selects a feature when splitting. Each random forest will output features with an important factor, then we rank the features after the average important factor for all forests and combine features of all levels according to each forest feature’s importance. In each level, the entire model is validated on the training set. Compared to most deep neural networks with fixed model complexity, the cascade forest adaptively determines its model complexity by terminating training when it is sufficient. This makes it suitable for training data at different scales. Finally, averaging across all trees in the same forest, and the class distribution for each forest is generated.

### The overall procedure of Kernel Principal Components based Cascade Forest (KPCCF)

KPCCF model is composed of two modules: firstly, using kPCA to reduce the high dimension of the input “large p, small n” data; secondly, using the cascade forest depth model to improve the model’s classification ability.

**Fig. 3:**
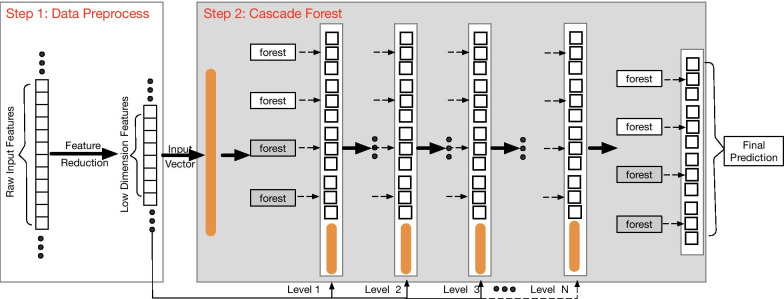
The overall procedure of KPCCF. Firstly, apply the RBF kernel function to adjust the datasets. Secondly, input the extracted outputs features of the previous stage into the cascade forest

The overall procedure of the KPCCF algorithm is shown in Fig. 3.

*Step One* Apply feature reduction in divided training set to adjust the datasets better suitable for disease classification prediction. Though not all categories are distinguished, it will still catch some similarity factors. By RBF kernel function, the unknown correlation high-dimensional data will be transformed into approximately linearly separable data.

*Step Two* The features extracted from the previous stage by kPCA method are fed to the cascade forest. Each layer of cascade forest is composed of multiple forests and will produce a class vector as its output. The class vector will connect with the former stage output, and then inputs the next layer. The next layer produces another class vector, which will further connect with an output produced by another branch of kPCA. This process continues until reaching the termination condition, such as achieving the expected accuracy or reaching the maximum number of layers. After getting the final class vector, calculate the average value for all kinds of possibilities and select the class with the maximum aggregated to be the final classification result.

KPCCF is a novel decision tree aggregation method, and its prediction accuracy is highly competitive with deep neural networks in a wide range of tasks. Besides, the deep forest is easier to train because it has fewer hyper-parameters than deep neural networks. Its performance is robust to hyper-parameter settings in different domains’ datasets, and it can get excellent performance even by using the default setting [30]. Another advantage is that the model complexity of the deep forest can be automatically determined for different training datasets, making the deep forest work well even on small datasets. Therefore, the advanced feature reduction makes the cascade forest algorithm much more suited for disease prediction.

## Results

In order to verify the proposed method, in this section, we tested the performances of various classifiers derived respectively from KPCCF and other state-of-the-art methods, including Decision Tree (DT) [45], standard ensemble method RF [21], the normal deep learning algorithm CNN [28], and the original deep forest model gcForest(DF) [27, 33, 34] on the four datasets shown in Table 1, and evaluated the results through classification accuracy.

### Experiment design and parameter settings

As the downloaded four microbiota datasets are composed of the most basic hierarchical species microbiome, we preprocess the datasets according to procedures in Fig. 1 and form the family level microbiota datasets. The newly-built ones are composed of a set of input features (various microbiota in the unit sample) and disease tags.

Since the division of the training set and the testing set largely affects the final model and parameter values, it is necessary to use as much data as possible to participate in the training of the model during model training. The LOO-CV (Leave-one-out cross-validation) method only uses 1 sample for testing at a time and uses other n-1 samples for training, which takes too long. Therefore, we use K-Fold Cross-Validation. Cross-validation (CV) is a common statistical analysis method used to verify the performance of classifiers. Firstly, divide all datasets into K parts, and take one of them as the testing set without repeating each time. Secondly, use the other K-1 parts as the training set to train the model. Then calculate the Accuracy and F1 values for the testing set, and K times of Accuracy are averaged to get the final Accuracy. The fundamental reason for the use of cross-validation is the limited sample number of data. In this case, using all data to train the model easily leads to overfitting. Such low bias and high variance results are not conducive to repeated experiments. When the model stability is low, increasing the value of K can get better results, but the computational overhead must be considered. As a practical application of medical diagnosis, the efficiency of providing decision support is also very important. Therefore, the k value cannot be set too large, which will easily lead to a decrease in calculation efficiency. As for the choice of k, sklearn uses 75% of the data set as the training set and 25% of the data set as the test set by default. And we also refer to articles [25] related to our research. Considering that the data in the medical field is unbalanced, to guarantee the testing set covers all the sample labels when the dataset is randomly divided, 1/4 of the data is proper to be selected as the testing set. Therefore, in the selection of K value, we choose 4-fold cross-validation as the experimental training test method. The results are evaluated through the prediction error and their square sum. For each dataset, the CV process has been conducted 20 times, and the average performance is evaluated as the final result. The samples in each dataset are randomly divided into 4 parts evenly. Each part of the samples is respectively used as a testing dataset and the remaining parts of the samples make up the training dataset. During each fold, the training dataset is fed into different classifiers to train the model, then the testing dataset is used to test the trained classifiers.

DT performs as the tree structure. It starts from the root node, then tests the corresponding feature attributes in each item to be classified, and selects the output branch according to its value until the leaf node is reached. The category stored by the leaf node is used as its result.

One of the improved bagging DT algorithms, RF, is a classifier using multiple decision trees to train and predict samples. Select the category with the most votes in the classifier’s voting results as the final classification result. For the random forest, the number of trees and the max-depth are tested with the grid searching method. We set them 100 and 2, respectively. All other parameters are left as default, such as max_features (default is auto), min_samples_split (default is 2), and min_samples_leaf (default is 1). We use the value of Gini impure to calculate properties and select the most appropriate node.

**Fig. 4:**
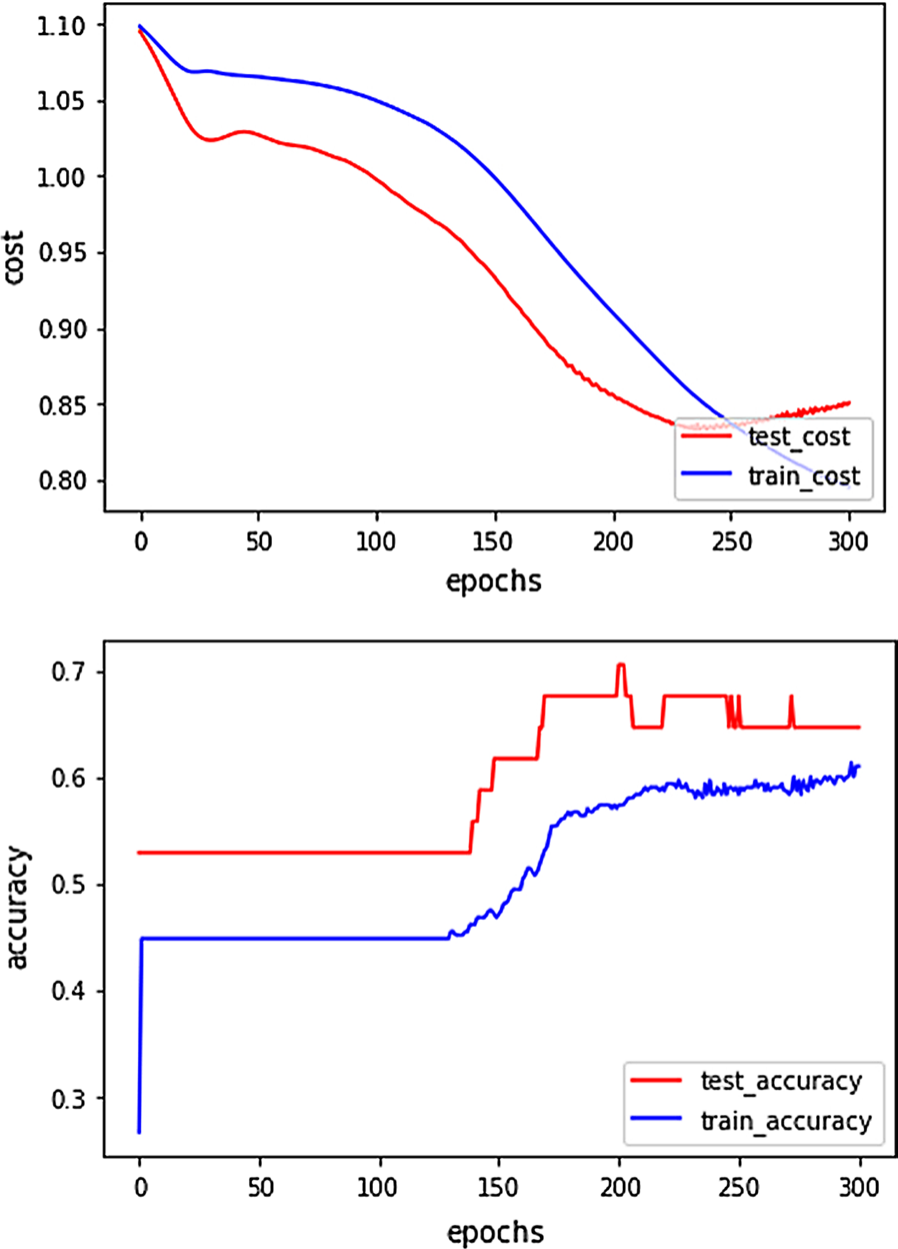
The loss and the accuracy of CNN in the different epoch of the ibd_papa dataset. The loss function is decreasing as epochs grow, and the accuracy outperforms consistently as dimension increases

In the model of CNN, the original input 2-dimension sample-feature data vector needs to be expanded to the 3-dimension, that is, turned from 2*2 to 1*2*2 to make the dimension conform to the model’s input. We decide how many hidden layers are best in the disease classification based on experimental tests, errors, and accuracy. 6 hidden layers are used in this study (including 3 convolution layers, 2 pooling layers, and 1 fully-connected layer). The multi-class classification of this experiment uses categorical cross-entropy as the loss function. By using the method of Stochastic Gradient Descent (SGD), recursively approximating the minimum deviation model, and using the chain derivation rule to deduct the nodes of the hidden layer, the ultimate goal is to make the loss of all training data as small as possible. The disease classification result is obtained at the output layer after the transformation of the hidden layer. The loss and the accuracy of CNN in different epoch are shown in Fig. 4. We can see that the loss function is decreasing as epochs grow, and the accuracy outperforms consistently as dimension increases. The loss value on the testing set starts to rise again after 200 epochs. Because the data is too small, the accuracy changes slowly in the early stages. Through the curve of accuracy, it can be found that the fit has also begun to appear after 200 epochs.

**Fig. 5:**
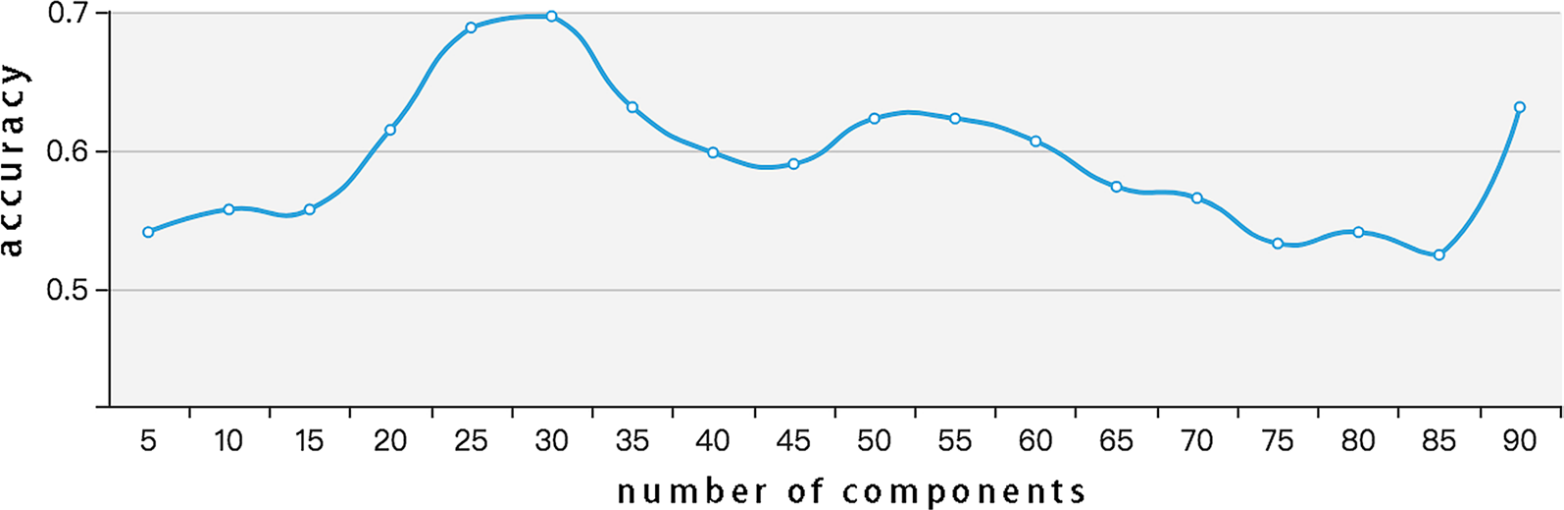
Accuracy of different principal components. We have varied the parameter number of components from 5 to 90 with the step size of 5. We abstract 30 principal components at the family level as it reaches their peak

For KPCCF training, suppose that the original 1-D microbiome input is of 100 raw features. In the feature reduction module, taking the cdi_schubert dataset at the family level as an example, each sample has 90 features. According to the number of the dataset features, we have varied the parameter number of components from 5 to 90 with the step size of 5, the accuracy is shown in Fig. 5. We can abstract 30 principal components at the family level as it reaches their peak. However, in the process of dimension reduction, the features number in our four datasets varies from 49 to 93 at the family level and from 142 to 255 at the genus level. We cannot find the number of features that can optimize the final accuracy and computing efficiency at the same time. As a result, we set the hyper-parameter “$n\_components$” as mle at last, which means the number of features will be automatically selected to meet the required percentage of variance. That is, the model will select a certain number of principal components features to reduce dimensionality according to the variance distribution of the feature, which we find can balance the final accuracy and the compute efficiency.

After feature reduction by kPCA, the transformed training set will then be used to train the 1st-grade of a cascade forest. These data will be used to train two random forests and two completely-random tree forests. Each forest contains 30 trees generated by randomly selecting a feature for a split at each node of the tree and growing tree until each leaf node contains only the same class of instances. If there are three classes to be predicted, then each of the four forests will produce a three-dimensional class vector. Thus, the next level of the cascade will receive augmented features. Compared to most deep neural networks with fixed model complexity, the cascade forest adaptively determines its model complexity by terminating training when it is sufficient. As a result, the KPCCF model has a few parameters to adjust.

### The performance comparison of various classifiers

**Fig. 6:**
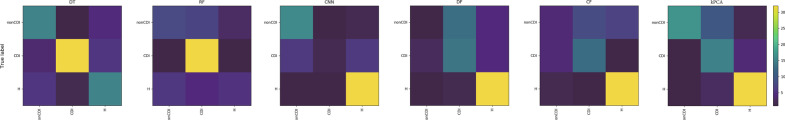
The confusion matrix of 3 classification. Above all, KPCCF has the best results for its diagonal color is the lightest

In this paper, every dataset has been tested 20 times in all 6 methods, DT, RF, CNN, CF, DF, and KPCCF, with the data being divided differently. And we take the average as their final results. Taking the cdi_schubert dataset as an example, the confusion matrix of one experiment by six algorithms is shown in Fig. 6. As we can see, various algorithms identify diseases with different sensitivity. DT and RF identify samples with CDI disease well, while CNN, DF, CF, and KPCCF algorithms can identify healthy samples well. Above all, KPCCF has the best results for its diagonal color is the lightest. In specific, KPCCF classifies 22 samples as nonCDI, while 8 of these are supposed to be CDI in reality. It predicts 17 to be CDI with 11 to be true. 45 of the training samples are diagnosed as healthy, and only one of them is wrong.

When the dataset is unbalanced, using accuracy measures to evaluate the classification performance is not enough, some other metrics, like “*precision*” and “*recall*”, or a combination of the two. In the multi-category problem, the F1 score is divided into two types, which are $Macra\ F1\ score$ and $Micro\ F1\ score$ respectively. The n-class classification problem is divided into n two-category evaluations, and the F1 score of each two classifications is calculated. As $Macra\ F1\ score$ is the average of n $F1\ scores$ and is heavily influenced by the small number of samples. The use of $Micro\ F1\ score$ is more reasonable in the case of uneven data samples.

We use accuracy (*Acc*), variance (*Var*), and *MicroF*1*score* as model evaluations. As can be seen in Table 2, the *Acc* and the $Micro\ F1\ score$ of the KPCCF algorithm are generally better than the other five existing algorithms. In all datasets, CNN always got the lowest accuracy except in the ob_goodrich dataset. It’s probably because the ob_goodrich dataset has a relatively larger dataset, while CNN is easier to over-fitting, especially when the datasets are extremely small like what is used in this article. When the sample number of the dataset is extremely small, such as the ibd_papa dataset, KPCCF showed an overwhelming advantage whose accuracy reached up to 0.57. It’s a 3-class classification problem with only less than 100 samples. And it has much more hyper-parameters to adjust. The predictions of CF and DF models are not very stable, while kPCA can improve the situation. It is also noticeable that the DF model performs well in the ibd_papa and ob_goodrich datasets but poorly in the other two datasets. This is because their features are relatively smaller compared to their samples’ size. Thus, by use of feature reduction method reasonably, cascade forest, which is a deep forest model, may produce sensible results on the datasets.

**Table 2:** The performance comparison of different models in disease identification

Disease	cdi_schubert			crc_baxter			ibd_papa			ob_goodrich		
	Acc	Var	F1	Acc	Var	F1	Acc	Var	F1	Acc	Var	F1
DT	0.66	0.043	0.68	0.40	0.035	0.41	0.48	0.091	0.48	0.39	0.021	0.39
RF	0.63	0.038	0.65	0.41	0.033	0.41	0.52	0.089	0.52	0.47	0.029	**0.48**
CNN	0.56	0.068	0.54	0.38	0.048	0.37	0.47	0.090	0.43	0.43	0.045	0.41
CF	0.67	0.053	0.69	0.40	0.042	0.4	0.53	0.082	0.54	0.46	0.026	0.44
DF	0.61	0.037	0.64	0.39	0.042	0.37	0.53	0.074	**0.57**	0.46	0.022	0.46
KPCCF	**0.69**	0.057	**0.71**	**0.43**	0.040	**0.48**	**0.57**	0.072	**0.57**	**0.47**	0.012	**0.48**

The result with the best performance is bold

**Fig. 7:**
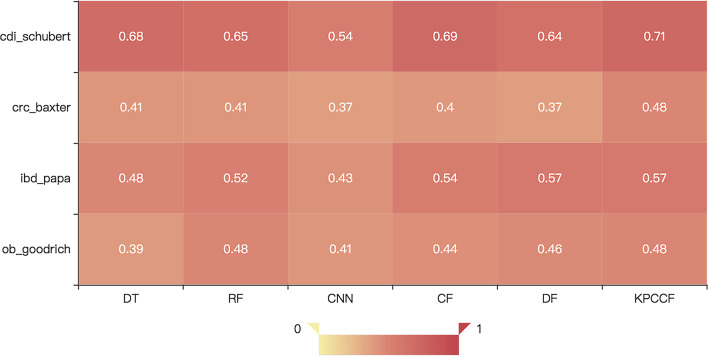
The $Micro\ F1\ score$ of 4 prediction results at the family level respectively. CDI disease is the most adaptive to classify

To more intuitively display the results in the table, we visualize some of the results. In the multi-class classification problem, the Micro F1 score is more accurate to measure the algorithms. The Micro F1 score of 4 prediction results at the family level respectively is shown in Fig. 7. In the thermal map, the darker the color, the larger the value. It can be easily found that CDI disease is the most adaptive to classification methods for its color is always much darker than other datasets. While CRC and OB diseases get prediction far from satisfied. That’s maybe because the diseases cannot be easily classified by our used microbiota data. The differences in the characteristics of the different labels are not as obvious as the other two groups. Diseases like obesity’s associations with the microbiome remain unclear require potential confounders, like host behavior and diet [20]. It needs further improvement if it is to be put into practical use. Either from the perspective of algorithms or from the perspective of microbiota flora processing.

The biggest advantage of KPCCF is that: (1) it has excellent performance even with a small amount of data as it’s the ensemble of RF, (2) it has fewer hyper-parameters compared to DNN, and (3) compared to the multi-scanning stage of DF, it discovers nonlinear high-order correlations between data and removes this correlation without knowing the relationships between microbiota community in advance.

## Discussion

### Extended study based on metadata

To explore the relationship between metadata and disease, here, we use four datasets by fusing their microbiota data and the metadata as the mixed datasets covering CDI, CRC, IBD, and OB diseases, and train the KPCCF diagnosis model again. Since the information in each metadata is different, we add age and gender features in the cdi_schubert and ibd_papa dataset, add BMI, age, and gender in the crc_baxter dataset, add age in the ob_goodrich dataset. Based on the mixed datasets, the various models are tested via similar settings as before. The performance of microbiota data only and mixed data fused with metadata is shown in Table 3. Results show that the concatenation improves the accuracy score in CRC, IBD, and OB. This means these three diseases may have a great relationship with samples’ gender, age, and other characteristics and simply concatenating them brings better results. The prediction accuracy of CRC and OB increased by 0.05 reaching 0.48 and 0.52 respectively, which is great progress. In specific, we find that older people are more likely to get sick in these three datasets. While there is no obvious relationship between CDI and age and gender for the predicted accuracy even decreases. However, the accuracy of CDI prediction still ranks highest.

**Table 3:** The prediction accuracy of microbiota data only and mixed data fused with metadata

Disease	Microbiota data	Mixed data
cdi_schubert	0.69	0.68
crc_baxter	0.43	0.48
ibd_papa	0.57	0.59
ob_goodrich	0.47	0.52

### Comparative study between the genus level and the family level microbiota

Using the family level will be more beneficial to the application as we analyzed before. To verify this, we compare the prediction accuracy of the genus level and the family level in all datasets. Similarly, the KPCCF model has been tested 20 times via the genus level dataset and family level dataset.

**Fig. 8:**
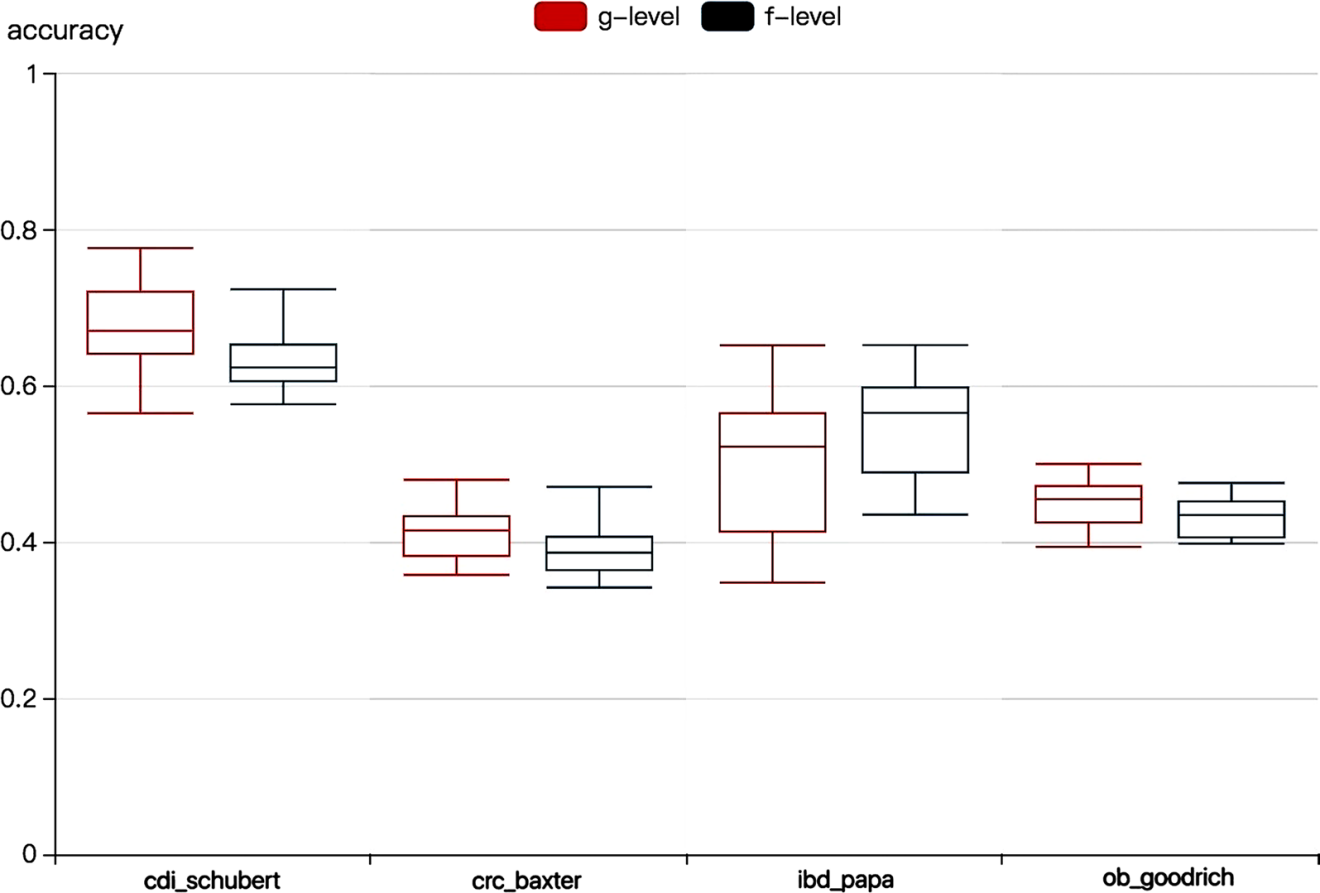
The accuracy of KPCCF prediction results at the genus level and the family level respectively. The family level performs more stable than the genus level

According to the comparison of KPCCF prediction results at the genus level and the family level respectively shown in Fig. 8, it is found that the algorithm has a certain variance on each dataset. Among them, the results of the first and third datasets fluctuate greatly, and the results of some extreme values deviate from the average. The results on the second and fourth datasets are relatively stable. In all of the four datasets, the family level performs more stable than the genus level. Most of the average accuracy is slightly reduced, but the results are better on the ibd_papa dataset. In medical diagnosis, the stability and the accuracy of the results are equally important. The former guarantees the reliability of the algorithm, while the latter guarantees the validity of the algorithm. The best result of the experiment is to compromise both reliability and validity. What’s more, the family level has fewer features, thus time-saving and further avoiding overfitting.

### Comparative study among various dimension reduction methods

**Fig. 9:**
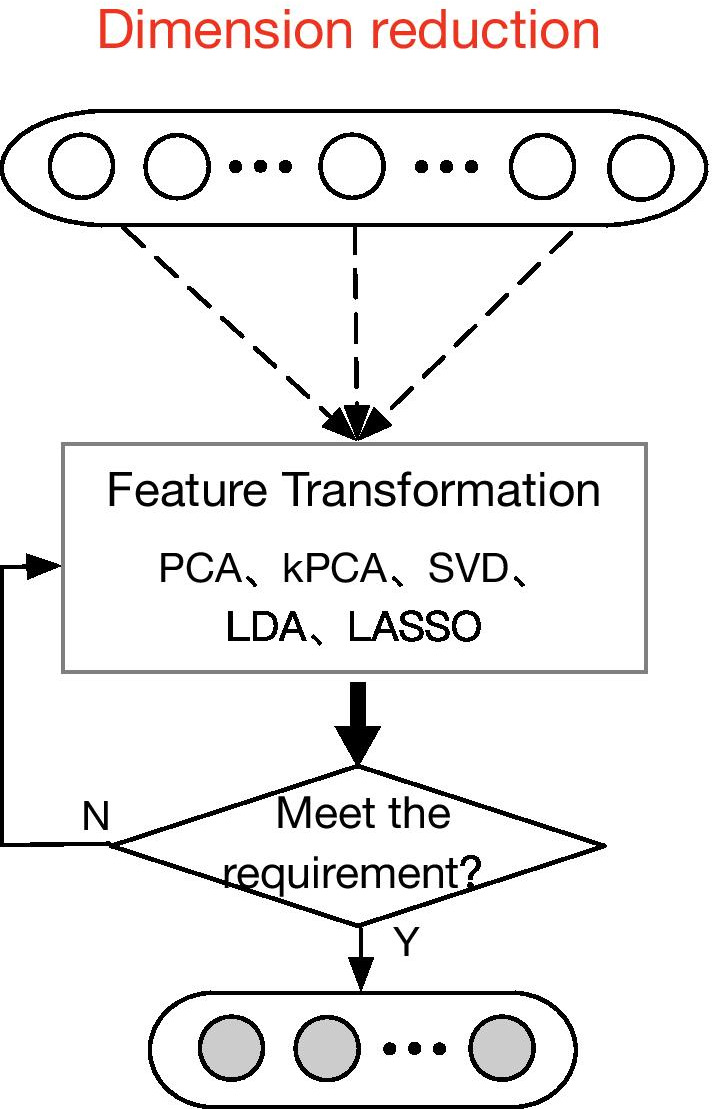
Process of comparative study among various feature reduction methods. We make a comparison between Principal Components Analysis (PCA), kernel Principal Components Analysis (kPCA), Singular Value Decomposition (SVD), Linear Discriminant Analysis (LDA), Least Absolute Shrinkage, Selection Operator (LASSO) dimension reduction methods

To validate the usefulness of our used kPCA method, by substituting it with Principal Components Analysis (PCA), Singular Value Decomposition (SVD), Linear Discriminant Analysis (LDA), Least Absolute Shrinkage, and Selection Operator (LASSO) dimension reduction methods, respectively, we conduct CV experiments with cascade forest on 4 datasets for comparison. The dimension reduction process is shown in Fig. 9.

PCA dimension reduction requires the largest *d* eigenvectors of the sample covariance matrix $X^{T}X$ and then using the matrix of the largest *d* eigenvectors to make low dimensional projection dimensionality reduction. SVD can also obtain the matrix of the largest *d* eigenvectors of the covariance matrix $X^{T}X$, but SVD has another advantage. SVD is especially effective when the sample size is large. In fact, PCA only uses the right singular matrix of SVD, but the left singular matrix can also be used for row number compression. In contrast, the right singular matrix can be used for the compression of the number of columns, that is, the feature dimension.

The principles of LDA and PCA are different. PCA is an unsupervised algorithm projected to the direction by the sort of data variance. The assumption is that the larger the variance, the more information there is. While for LDA, it is projected after the selection of the smallest intra-class variance and the largest variance between classes. Considering specific purposes and scenarios, in classification problems, the feature reduction criteria for LDA are more reasonable.

LASSO raised the problem that the ridge regression cannot be parameterized, and it can select parameters by parameter reduction to achieve dimension reduction. The penalty term is a norm, and some parameters can be forced to 0 to achieve the purpose of parameter selection.

**Fig. 10:**
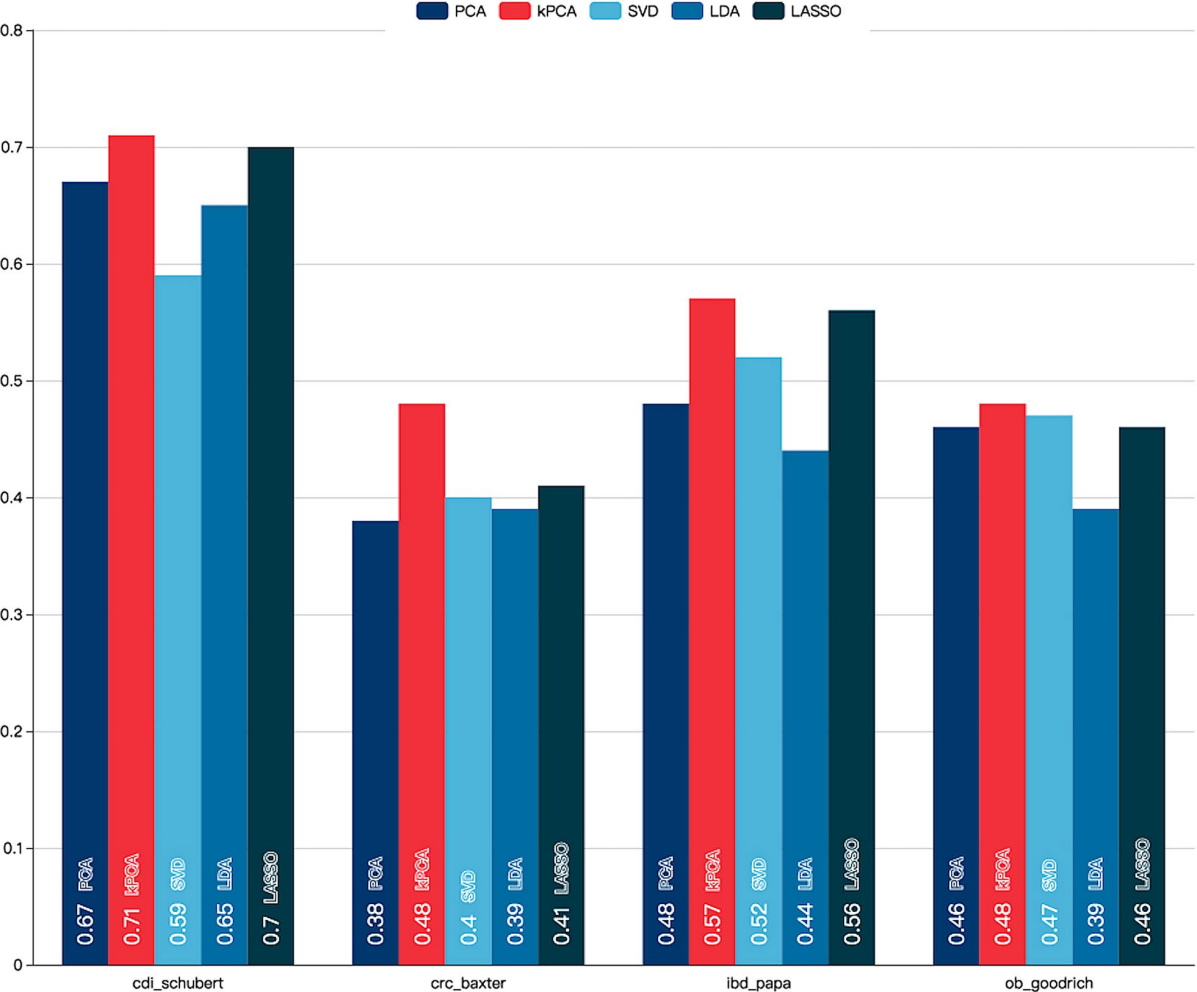
The $Micro\ F1\ score$ of 5 feature reduction models in each dataset. The kPCA model in red color has the most prominent performance among these 5 methods with $Micro\ F1\ score$

We use the $Micro\ F1\ score$ to evaluate different feature reduction models. The $Micro\ F1\ score$ of 5 algorithms prediction results in each dataset is shown in Fig. 10. As we can see, the kPCA model in red color has the most prominent performance among these 5 methods with $Micro\ F1\ score$ 0.71, 0.48, 0.57, and 0.48 respectively. LASSO’s performance ranks in second place with $Micro\ F1\ score$ 0.7, 0.41, 0.56, and 0.46 respectively, which are very close to the best performance on most datasets. While the $Micro\ F1\ score$ in PCA, SVD, and LDA fluctuates in different data sets.

## Conclusion

Considering the genus level vast microbiota species and the difficulty of sequencing, it is more advantageous to make a predictive analysis at the family level. In this work, we propose a KPCCF model to solve the problem of disease identification based on the family level microbiome. To prove the superiority of the proposed model, we conduct the multi-class classification experiment on four different real microbiota datasets and compare its performance with other state-of-the-art algorithms, including DT, RF, CNN, CF, and DF algorithms. The results confirm that our improved cascade forest model KPCCF performs comparatively better, while cascade forest can adapt to larger datasets and get better results. Furthermore, we carry out the extended study by combining the microbiota data with the corresponding metadata and finding the insertion of the metadata that can effectively improve the accuracy of disease identification. In the end, we explore different mainstream feature reduction algorithms and find kPCA is the best selection for our microbiota datasets.

The contributions of our work are summarized as: (1) we introduce the kPCA method into the cascade forest algorithm, which can both effectively reduce the feature dimension and improve the classification accuracy; (2) instead of the traditional two-class disease diagnosis problem, we explore a multi-class classification model to solve the disease identification problem with more than three disease states; and (3) in practical application, we only utilize numbers of microbiota at the family level for supervised learning and find ways to improve disease identification accuracy, which is a great challenge. However, due to the difference between individuals, when there is a small number of samples, the trained model may lack generalization ability. In our future works, we will focus on improving the generalization ability of our KPCCF model. One feasible way is using transfer learning to construct more samples from the samples with different diseases or health states.

## Data Availability

The datasets used in this paper are from the standardized database of the human intestinal microbiome study: MicrobiomeHD, https://zenodo.org/record/1146764#.XDv1O_xS9sN.

## Abbreviations

HIV: Human Immunodeficiency Virus
DNA: DeoxyriboNucleic Acid
RNA: RiboNucleic Acid
D: Domain
K: Kingdom
P: Phylum
C: Class
O: Order
F: Family
G: Genus
S: Species
CDI: Clostridium Difficile Infection
CRC: ColoRectal Cancer
IBD: Inflammatory Bowel Diseases
OB: OBesity
CD: Crohn’s Disease
UC: Ulcerative Colitis
OW: OverWeight
OTU: Operational Taxonomic Units
KPCCF: Kernel Principal Components based Cascade Forest
RF: Random Forest
SVM: Support Vector Machine
DL: Deep Learning
DNN: Deep Neural Network
CNN: Convolutional Neural Networks
DF: Deep Forest
kPCA: kernel Principal Components Analysis
RBF: Radial Basis Function
CV: Cross-Validation
SGD: Stochastic Gradient Descent
PCA: Principal Components Analysis
SVD: Singular Value Decomposition
LDA: Linear Discriminant Analysis
LASSO: Least Absolute Shrinkage and Selection Operator
